# Association between social and built environment characteristics and maternal mortality in 340 Latin America cities: an ecological study from the SALURBAL study

**DOI:** 10.1136/bmjph-2024-002437

**Published:** 2026-01-14

**Authors:** Camila Teixeira Vaz, Uriel M Silva, Ana Ortigoza, Monica Serena Perner, Marcio Alazraqui, Ariela Braverman-Bronstein, Duane Alexander Quistberg, Amélia Augusta de Lima Friche, Waleska T Caiaffa

**Affiliations:** 1 Federal University of São João del-Rei, Divinópolis, Brazil; 2 Federal University of Minas Gerais, Belo Horizonte, Brazil; 3 Department of Social and Environmental Determinants for Health Equity, Pan American Health Organization, Washington, District of Columbia, USA; 4 Universidad Nacional de Lanus, Lanus, Buenos Aires, Argentina; 5 Instituto de Salud Colectiva, Universidad Nacional de Lanus, Lanus, Buenos Aires, Argentina; 6 Institute for Community Health, Malden, Massachusetts, USA; 7 Drexel University Dornsife School of Public Health, Philadelphia, Pennsylvania, USA; 8 Medicine, Federal University of Minas Gerais, Belo Horizonte, Brazil

**Keywords:** Public Health, Environmental Medicine, Epidemiologic Factors

## Abstract

**Introduction:**

Maternal mortality ratio (MMR) is a key indicator of maternal health, but heterogeneity across cities has been infrequently explored. We aimed to investigate variation in MMR across cities and the association between social and built environment features of urban areas and MMR in Latin American countries.

**Methods:**

This ecological study used harmonised data from the Salud Urbana en América Latina study comprising 340 cities across eight countries (Argentina, Brazil, Chile, Colombia, Costa Rica, Guatemala, Mexico and Panama). MMRs were calculated for each city using vital statistics registries for the years 2012–2016 and a correction factor applied for under-reporting of maternal deaths. Predictors of interest included city social environment characteristics (living conditions, services provision and population educational attainment), built environment characteristics (isolation of urban development, population density and mass transit availability) and total population. Mixed negative binomial regression models with country-level random intercepts were used. Rate ratios (RRs) were estimated.

**Results:**

MMR was 47.3 maternal deaths (SD 25.3) per 100 000 live births. Cities with better living conditions and with better services provision had lower MMRs (RR=0.89 per SD, 95% CI=0.82 to 0.97; and RR=0.92 per SD, 95% CI=0.87 to 0.97, respectively), while those with higher urban isolation and population density had higher MMRs (RR=1.07 per SD, 95% CI=1.03 to 1.13; and RR=1.11 per SD, 95% CI=1.04 to 1.19, respectively).

**Conclusions:**

We found that better living conditions, service provision and mass transit availability were associated with lower MMR, whereas higher population density and urban isolation were associated with higher ratios. These findings suggest that multisectoral urban policies aimed at improving social conditions, infrastructure and mobility may help reduce maternal mortality in Latin American cities, contributing to improving maternal health and advancing health equity.

**WHAT IS ALREADY KNOWN ON THIS TOPIC**

- Maternal mortality remains high in Latin America, even after the substantial progress observed in recent decades.
- Previous studies have mostly focused on individual or healthcare factors, with limited evidence on how urban environments affect maternal mortality across cities.

**WHAT THIS STUDY ADDS**

- Using harmonised data from 340 cities in 8 Latin American countries, this study shows that better living conditions, services provision and mass transit availability are associated with lower maternal mortality ratios, while higher landscape isolation and population density are associated with higher ratios.
- City-level social and built environment features account for much of the variation in maternal mortality observed between cities within countries.

**HOW THIS STUDY MIGHT AFFECT RESEARCH, PRACTICE OR POLICY**

- Improving urban living conditions, basic services and public transport while reducing landscape isolation and population density could help decrease maternal mortality in urban areas in Latin America.
- Findings highlight the importance of multisectoral urban planning combined with health policies to address persistent maternal health inequalities in the region.

## Introduction

 Worldwide, maternal mortality is unacceptably high with an estimated 260 000 maternal deaths in 2023, most of which (92%) were in low-income and middle-income countries (LMICs).^1^ Deaths during pregnancy, childbirth and postpartum have devastating social consequences as they represent not only the loss of a family member but can also increase mortality among children whose mothers died during or after their births, as well as a reduction in household income.^2^ This can perpetuate the cycle of poverty in communities across generations.^2^

From 2000 to 2023, maternal deaths have decreased by 40%; despite this, 700 women still die each day from largely preventable causes before, during and after the time of giving birth.^1^ Latin American and Caribbean (LAC) experienced the smallest percentage reduction in maternal mortality ratio (MMR) globally (16.8%).^1^ In the region, MMR declined substantially between 2000 and 2015 but increased again between 2016 and 2023, reaching a point estimate of 77 maternal deaths per 100 000 live births by the end of the period.^1^ This estimate included three years of observation (2020-2022), during which the COVID-19 pandemic was ongoing.^1^ Although it is plausible that the COVID-19 pandemic contributed to the trend seen in the MMR estimates from 2016 to 2023,^1^ this increasing trend in maternal mortality in the region may indicate that for achieving further reductions, it will be necessary to address drivers of mortality related to social inequalities in the population, once preventable maternal deaths due to healthcare have been addressed.^3^

Reducing maternal mortality has been a major global concern and a global health priority,^1^ since it has been estimated that 88%–98% of maternal deaths are preventable.^4^ In addition, the MMR estimates in the LAC region differ between countries and across cities within countries in the region, which highlight inequalities in MMR that cannot be attributed only to biological or individual differences.^5^ These differences have been attributed to a particularly vulnerable environment for women that results not only from poorer access to healthcare, lack of coordination between healthcare providers or variation in hospital quality of care during childbirth, but may also be due to, or exacerbated by, a greater exposure to environmental and social conditions hazardous to health.^6^

Social inequalities in cities linked to maternal mortality have been infrequently examined. A few studies analysed social and built environment features at the subnational level, relating maternal deaths to poor sanitation,^7^ geographic isolation,^7^ living in deprived areas,^8 9^ racial disparities, low income and health insurance.^10^ Studies exploring the relationship between poor sanitation and maternal death have found that poor hygiene at the time of delivery could lead to sepsis.^11^ Moreover, the higher MMR in more deprived neighbourhoods has been linked to late antenatal care and insufficient understanding of warning symptoms.^8^

The LAC region is considered the most urbanised and unequal region in the world, with 80% of the population living in urban areas with high levels of social, economic and environmental inequalities.^12^ The rapid and unplanned expansion of cities in LAC has resulted in a large proportion of the urban residents living under conditions of overcrowding, insecure property rights, deficient urban and social services and exposure to crime and violence, among other socioeconomic problems.^12^

Investigating how characteristics of cities affect maternal deaths is important to identify actions and policies to improve maternal health and promote health equity in the context of a rapidly urbanising world. Using data from a unique multinational study, the Salud Urbana en América Latina Study (SALURBAL), we aimed to quantify differences in MMR across a wide range of cities in Latin America and examine how key features of urban environment are related to this indicator.

## Methods

This is an ecological study that used data from SALURBAL, Urban Health in Latin America Project, an international collaboration that studies how urban environments and urban policies impact the health of city residents throughout Latin America.^13^ The SALURBAL project has compiled and harmonised data on health as well as social and built environments from 371 large cities (population ≥100 000 in 2010), in 11 countries.^14^ A SALURBAL ‘city’ is defined geographically as single or clusters of administrative units (ie, municipios, comunas, partidos, delegaciones, cantones or corregimientos) that encompassed the urban extent of the city in 2010 using satellite imagery. More details are available elsewhere.^14^ For this study, we included 340 cities from 8 countries: Argentina, Brazil, Chile, Colombia, Costa Rica, Mexico, Peru and Panama that had available vital statistic registries for the period under study.

According to the International Statistical Classification of Diseases and related health problems (ICD), a maternal death is defined as ‘the death of a woman while pregnant or within 42 days of termination of pregnancy, irrespective of the duration and site of the pregnancy, from any cause related to or aggravated by the pregnancy or its management but not from unintentional or incidental causes’.^15^ The WHO has defined MMR as ‘the number of deaths during a given time period per 100 000 live births during the same time period’.^1^ For this study, the outcome, MMR, was defined as the ratio between the ICD-defined maternal death counts and the number of live births, both of which were aggregated across the entire sample period (years 2012–2016) for each city.

We obtained data on maternal deaths from the vital registration systems of each country. These deaths were identified using the ICD version 10 codes O00–O99. Capturing these deaths from civil registries and from ICD codes may lead to underestimation of maternal deaths, and therefore, a correction factor is typically used to account for maternal deaths.^1^ We used two undercounting correction factors. For the main analyses, we used an ensemble of general death distribution methods described elsewhere, developed at the city level, specifically for the SALURBAL project,^16^ and in a sensitivity analysis we used a correction factor derived from the Bayesian Civil Registration and Vital Statistics (CRVS) adjustment model, developed specifically for maternal deaths, by the WHO, UNICEF and UNFPA, at national level.^1^

We explored several characteristics of cities hypothesised to be related to MMR based on prior literature on how cities develop and grow, how they organise transportation and land use, and how they manage access to quality housing and other resources that can impact the health outcomes of LAC residents.^12 13 17^ Social environment indicators at city level were obtained from harmonised variables in national censuses of each country.^14^ Three domains of indicators (living conditions, services provision and educational attainment) were identified using a principal component analysis,^3^ each corresponding to conceptually distinct aspects of the social environment, and all may be related to MMR through various mechanisms including behaviours, stressors and environmental exposures. For each of the domains, a score was defined as the average of the Z-scores of the indicators in that domain. Higher score values imply better social environment conditions.^3^ Details on the scores’ components and the corresponding data sources used are described in online supplemental material.

City built environment features included in the analysis were isolation of urban development, population density and mass transit availability. Isolation is defined as the mean distance (in metres) to the nearest urban patch within the geographic boundary, weighted by the area (in squared metres) of each patch. Population density was defined as the population per square kilometre in all of the urban patches inside the geographic boundary. Mass transit availability was based on the presence of passenger rail and bus rapid transit (BRT) networks in the city. These indicators capture city development, walkability and mobility, respectively, and they may be related to MMR through mechanisms involving access to services, including access to health services, since they can represent geographic barriers, especially for women living in peripheral areas of large urban centres.^18^

Total population was obtained from intercensal projections based on the national censuses of each country and was defined as the average of the population over the 2012–2016 period, for each city. A full description of variable definitions and data sources is provided in online supplemental Table S1.

### Patient and public involvement

Patients or the public were not involved in the design, conduct or reporting of this ecological study using aggregated vital statistics data.

### Statistical analysis

Descriptive analyses were performed by assessing the means and SDs of MMR and city characteristics by country. We further explored the distribution of MMR by producing boxplots for each country, and by analysing country-specific medians and IQRs. We also computed the correlation matrix of all the exposures (online supplemental figure S1l).

To assess variability in MMR between cities and countries, we fitted a multilevel linear model with the logarithm of MMR (added by 1 to ensure that the logarithm was properly defined) as the outcome and including a random intercept at the country level. The variability across levels was then evaluated by computing the percentage of the total variance corresponding to each level. Further details on this model can be found in the online supplemental material.

Finally, to investigate the association between city characteristics and MMR, we used generalised linear mixed models with the negative binomial distribution and robust standard errors. Four models were fitted: Model A included each exposure separately; Model B included all social environment measures jointly; Model C included built environment and population measures jointly and Model D included all measures. All exposures were standardised to have a mean of 0 and an SD of 1, prior to modelling to allow direct comparison of estimated effects, with city size also being log-transformed prior to standardisation. To have city-level MMR as the outcome, for each model we chose city maternal deaths as the dependent regression variable and used an offset equal to the logarithm of the corresponding number of live births, which was first multiplied by the undercounting correction factor and divided by 100 000. Rate ratios (RRs), therefore, represent the change in city MMR associated with a 1-SD higher value of the exposure. Each model also included a country-level random intercept. For model D, we computed variance inflation factors (VIFs) for each exposure to assess possible collinearity issues. Specific details on models A–D can be found in the online supplemental material. We also visually assessed potential non-linear associations between population density and log-MMR using a scatterplot with locally estimated scatterplot smoothing (LOESS) (online supplemental figure S2).

In a sensitivity analysis, we re-estimated models A–D using the Bayesian CRVS correction factor proposed by WHO.^1^ All analyses were conducted with the package glmmTMB in the R software environment, V.4.3.

## Results

Overall, the average urban MMR without correction across all countries was 47.3 per 100 000 live births (SD=25.3). The country with the lowest average of urban MMR was Chile (mean=22.38, SD=13.07 per 100 000 live births) and the one with the highest average of urban MMR was Colombia (mean=66.40, SD=35.51 per 100 000 live births) (online supplemental Table S2). To illustrate this geographic variation across the 340 cities, we provide a city-level map of MMR in online supplemental figure S3.

After accounting for the correction factors, the overall urban MMR increased by a value of 3.2 with the SALURBAL correction and by 4.6 with the CRSV correction. MMRs were similar regardless of correction in Argentina, Chile and Guatemala, but changed substantially when corrected in Colombia, Costa Rica and Panama (online supplemental Table S2).

Table 1 shows the distribution of exposures by quartiles of MMR. For social environment measures, we found that services provision scores were lower in higher quartiles of MMR. No pattern was found for the other two social environment measures. For the built environment exposures, isolation was lower in cities with the lowest quartile of MMR. Population density was higher in cities with higher MMR.

**Table 1:** Distribution of exposures variables by quartiles maternal mortality ratio (MMR)

Variables	Overall	MMR Q1	MMR Q2	MMR Q3	MMR Q4	P value
Number of cities	340	–	–	–	–	–
Outcome						
MMR (100 000 live births)	47.31	20.55	36.47	50.74	81.48	<0.001
	(25.31)	(7.91)	(3.62)	(5.13)	(21.13)	
Social environment exposures						
Living conditions	0.24	1.19	−0.67	0.04	0.38	<0.001
	(2.54)	(1.87)	(2.62)	(2.60)	(2.65)	
Services provision	0.15	0.76	0.63	0.19	−0.99	<0.001
	(1.76)	(1.42)	(1.32)	(1.56)	(2.10)	
Population educational attainment	−0.50	−0.41	−0.58	−0.30	−0.69	0.319
	(1.30)	(1.18)	(1.40)	(1.28)	(1.31)	
Built environment exposures and total population						
Isolation (patches/100 ha)	97.18	88.96	100.01	95.97	103.77	0.033
	(41.62)	(30.23)	(35.82)	(45.37)	(51.14)	
Population density (hab./km^2^)	7228	5825	6973	7532	8582	<0.001
	(3915)	(1717)	(3918)	(4252)	(4635)	
Mass transit availability (%)	14.41	12.94	10.59	22.35	11.77	0.530
	(35.17)	(33.77)	(30.95)	(41.91)	(32.41)	
Total population (mi. hab.)	0.80	0.58	0.86	1.13	0.66	0.021
	(2.08)	(1.00)	(1.94)	(3.21)	(1.47)	

**Figure 1:**
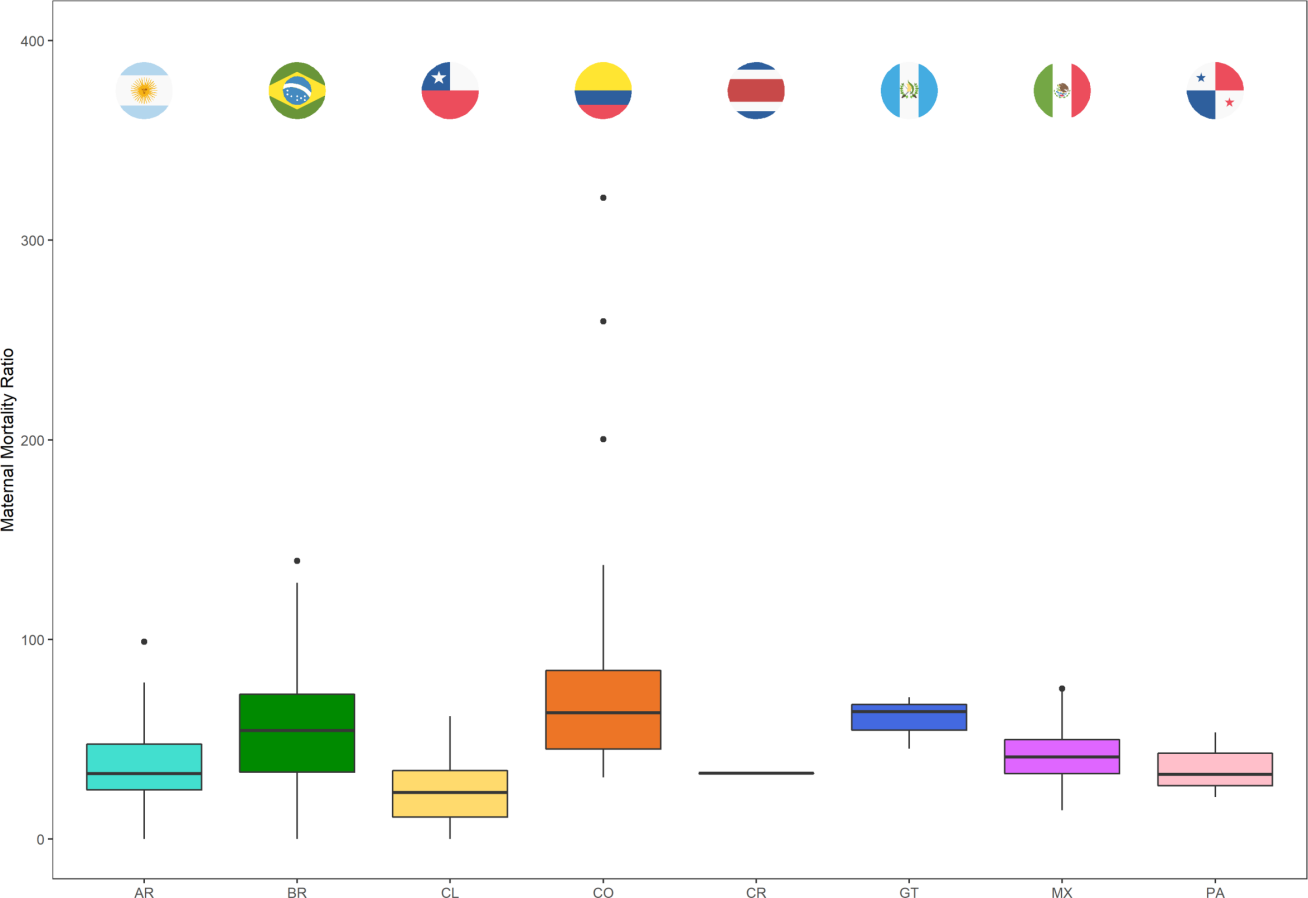
Boxplots of city-level maternal mortality ratios (MMR) across countries. AR, Argentina; BR, Brazil; CL, Chile; CO, Colombia; CR, Costa Rica; GT, Guatemala; MX, Mexico; PA, Panama.

Figure 1 shows the distribution of MMRs across cities by country, showing large heterogeneity both between and within countries. In the linear model decomposing the variance of MMRs across cities and countries, most of the variability was found to be between cities within countries (within country variance proportion=73.8% and between country variance proportion=26.2%).

Table 2 shows MMR RR associated with a 1 SD higher value of social and built environment features in cities. When examining each city feature as a single exposure (model A), cities with better social environment had lower MMR: RR=0.78 (95% CI 0.73 to 0.84) for better living conditions, RR=0.86 (95% CI 0.82 to 0.90) for better services provision and RR=0.91 (95% CI 0.87 to 0.96) for higher population educational attainment. Presence of mass transit was also associated with lower MMR (RR=0.96, 95% CI 0.92 to 0.99), while higher isolation RR=1.12 (95% CI 1.07 to 1.17) and higher population density were associated with higher MMR RR=1.12 (95% CI 1.05 to 1.20).

**Table 2:** RRs of city maternal mortality ratios associated with a 1 SD higher value of social and built environment features

Variables	Model A		Model B		Model C		Model D	
	RR (95% CI)	P value	RR (95% CI)	P value	RR (95% CI)	P value	RR (95% CI)	P value
Social environment features								
Living conditions	0.79 (0.73 to 0.85)	<0.001	0.87 (0.80 to 0.96)	0.004			0.90 (0.82 to 0.98)	0.014
Services provision	0.86 (0.82 to 0.90)	<0.001	0.91 (0.86 to 0.97)	<0.001			0.92 (0.87 to 0.97)	0.003
Pop. educ. attainment	0.91 (0.87 to 0.96)	<0.001	0.97 (0.92 to 1.02)	0.277			0.98 (0.93 to 1.04)	0.523
Built environment features and total population								
Isolation	1.12 (1.07 to 1.17)	<0.001			1.11 (1.06 to 1.17)	<0.001	1.07 (1.03 to 1.13)	0.003
Population density	1.12 (1.04 to 1.20)	0.002			1.13 (1.06 to 1.21)	<0.001	1.11 (1.04 to 1.18)	<0.001
Mass transit availability	0.95 (0.92 to 0.99)	0.021			0.94 (0.89 to 0.99)	0.029	0.95 (0.90 to 1.00)	0.049
Total population	0.97 (0.93 to 1.01)	0.139			1.02 (0.97 to 1.09)	0.421	1.04 (0.98 to 1.10)	0.204

All models are negative binomial regressions with maternal mortality ratios at the outcome, constant dispersion parameter and country-level random intercepts. A logarithmic link was used, and all exposures were standardised to have a mean of 0 and an SD of 1.

Model A: univariate associations.

Model B: multivariate (social environment exposures only) associations.

Model C: multivariate (built environment exposures and total population only) associations.

Model D: full model.

RR, rate ratio.

When exploring all of the social environment exposures together (model B), higher scores of living conditions and services provision remained statistically associated with lower MMR, although the associations were slightly attenuated: RR=0.87 (95% CI 0.79 to 0.95) for living conditions and RR=0.91 (95% CI 0.86 to 0.97) for services provision; the association of population educational attainment with MMR was no longer statistically significant (table 2).

In model C, which includes all the built environment exposures and total population jointly, the availability of mass transit remained negatively associated with MMR (RR=0.94 per SD, 95% CI 0.90 to 0.99), and isolation and population density remained positively associated with MMR, with point estimates being very similar to those observed in model A (table 2).

In the fully adjusted model (model D), higher scores of living conditions and services provision remained associated with lower MMR (RR=0.89 per SD, 95% CI 0.82 to 0.97; and RR=0.92 per SD, 95% CI 0.87 to 0.97, respectively), while isolation and population density remained associated with higher MMR (RR=1.07 per SD, 95% CI 1.03 to 1.13; and RR=1.11 per SD, 95% CI 1.04 to 1.19, respectively), with point estimates being very similar to those observed in model B. The association of mass transit availability with MMR was marginally significant (RR=0.95 per SD, 95% CI 0.90 to 1.00) (table 2).

Regarding model goodness-of-fit and diagnostics, marginal and conditional pseudo-R2 for models B, C and D were, respectively: 52.7% and 16.0%; 49.1% and 14.6%; and 54.4% and 27.0%. VIFs were below 5 for all exposures in model D, indicating no collinearity issues (online supplemental figure S4). Negative binomial overdispersion parameter values for models B, C and D were 0.0836, 0.0892 and 0.0730, respectively.

In the sensitivity analyses using the CRVS correction factor, in the full model (model D) living conditions, services provision and mass transit availability were also negatively associated with MMR, and isolation and population density were also positively associated with MMR, with point estimates being very similar to those observed in the main analyses (online supplemental Table S3). Therefore, no qualitative changes in the results were observed by using the CRVS correction factor instead of the SALURBAL one used for the main analyses.

## Discussion

We investigated the distribution of MMR across 340 cities of 100 000 inhabitants or more in eight countries of LAC, and its association with city social and built environment features and total population. Although countries differed in MMR, most of the variability (almost 74%) was between cities within countries. We found that higher levels of city living conditions, services provision and mass transit availability were associated with lower MMR. On the other hand, higher levels of isolation and population density were associated with higher MMR. This large heterogeneity in MMR observed across cities (even within the same country) and the associations we found highlight the importance of examining city-level factors as predictors of MMR.

Other studies from LAC^19^,^21^ and from high-income countries^8^ have showed substantial variability in MMR both within and between countries, as well as an association between MMR and socioeconomic indicators,^10 22^ in the same direction and magnitude of our findings. The lower socioeconomic scores we used in this work can be considered markers of social marginalisation^23^ and can represent an accumulation of heterogeneous risk factors present within these cities.^24^ City socioeconomic conditions could be linked to higher MMR through economic and cultural barriers in access to prenatal care, which is crucial in preventing complications that may lead to a maternal death.^25^ These socioeconomic indicators may affect reproductive outcomes, especially maternal mortality, by modelling a wide range of individual-level economic opportunities and risk behaviours.^24^ For example, better education yields higher socioeconomic status which in turn benefits maternal mortality, enhances women’s knowledge of health problems, increases awareness of the health services availability and accessibility, enhances female decision-making power and produces changes in household dynamics.^24 26^ In addition, city shared social environment features may influence unhealthy risk behaviours through common cultural norms and beliefs, which in turn are associated with maternal mortality.^24^ Moreover, other SALURBAL studies have found similar associations between social environment and other maternal and child outcomes such as infant mortality^3^ and adolescent birth rate,^27^ showing the interconnection of child and women’s health and how similar urban environments may impact these interrelated outcomes also similarly.

Higher isolation of urban development and lack of mass transit availability were associated with higher MMR. These measures are related to geographical barriers to mobility and geographical access to healthcare.^7 28^ Improving contextual city aspects is therefore an important possible target to reduce geographical barriers to healthcare, since most maternal deaths could be prevented with timely medical treatment. In addition, higher levels of population density were associated with higher MMR. A greater number of people per square kilometre can impact access to services and can lead to disordered urban environment, creating demands that cities are unable to face, resulting in higher MMR.

Our study was unique in analysing compiled and harmonised data on urban environments and MMR across more than 300 cities in Latin America, representing a wide variety of social and economic conditions. We were able to describe heterogeneity in MMR across cities and study how a range of city-level factors are related to MMR. Although other studies have focused on MMR in urban areas, to our knowledge, this is the first investigation examining the influence of both social and built environments and total population on MMR at the city level, across multiple cities and countries in Latin America. Some studies have documented associations of social and economic factors with MMR at a more disaggregated level, such as states or communities/neighbourhoods, but they were typically restricted to a single country. Few studies have examined these factors at the level of cities, and the study of city-level factors is especially relevant to the development of local interventions. National averages often hide differences at the local level, and these disparities can be strongly associated with socioeconomic indicators.^21^

Some limitations need to be considered when interpreting our results. Our analysis was ecological, so we could not account for individual-level factors such as education or income, and the cross-sectional nature of the study does not allow us to draw causal inferences and confounding remains a possibility. Analysis on MMR should account for omission in maternal deaths, since proper identification of which deaths are ‘maternal deaths’ remains challenging, particularly in developing countries, but this is a problem even in high income countries with complete and accurate vital registration systems.^29 30^ The main challenge stems from failure to report that the decedent was pregnant or had a recent pregnancy. To partly address underreporting, we included two correction factors: one developed by SALURBAL for all deaths,^16^ and another derived by the UN Population Division for maternal deaths,^1^ with similar results. However, these factors may not fully capture the impact of underreporting and, especially, heterogeneities by city. We also acknowledge that there was a time discrepancy between the year when exposures were collected to the year that the outcome was collected (2012–2016). Measures of public transportation were limited to passenger rail and BRTs. These have advantages over traditional buses, but they do not fully capture transit availability. Our analyses did not include any variable that characterised healthcare access or utilisation of health facilities as an adjustment variable. Access to (and use of) health services to receive antenatal care remains particularly unequal, and women from more disadvantaged areas tend to have inadequate care in terms of required number of visits and quality.^25 26^

## Conclusions

In rapidly urbanising LMICs, it is urgent to identify which urban policies are necessary to improve population health. We provided evidence that better city living conditions, services provisions and mass transit availability were related to lower MMR, while higher levels of landscape isolation and population density were related to higher MMR. Understanding the context-specific factors that may be related to maternal health is paramount to achieving the Sustainable Development Goals for maternal mortality. Our findings highlight the importance of prioritising multisectoral urban policies and interventions related to improving housing, sanitation, mass transit, isolation and population density in order to improve maternal health and decrease health inequity in the region. Our results highlight the potential role of city-level policies in improving these urban features and therefore reducing MMR.

## Supplementary material

10.1136/bmjph-2024-002437 online supplemental file 1

10.1136/bmjph-2024-002437 online supplemental figure 1

10.1136/bmjph-2024-002437 online supplemental figure 2

10.1136/bmjph-2024-002437 online supplemental figure 3

10.1136/bmjph-2024-002437 online supplemental figure 4

## Data Availability

Data are available on reasonable request.
