# Supplementary material for: Association between social and built environment characteristics and maternal mortality in 340 Latin America cities: an ecological study from the SALURBAL study
Source: BMJ Public Health. 2026 Jan 14;4(1):e002437. doi: 10.1136/bmjph-2024-002437 (PMC12815145; doi:10.1136/bmjph-2024-002437)
Supplement: online supplemental file 1 [file bmjph-4-1-s001.docx]

**Supplementary Material to “Association between social and built environment characteristics and maternal mortality in 340 Latin America cities: an ecological study from the SALURBAL study”**

**Negative Binomial GLMM specification**

The “fully adjusted” model in the paper corresponds to

$$Y_{ij}|X_{ij}\sim Negative Binomial\left( \mu_{ij}, \phi\right),$$

where $Y_{ij}$ is the maternal death count at the $i$th city and $j$th country, $i=1, \ldots, I$, $j=1, \ldots, J$ (there are a total of $I$ = 340 cities and $J$ = 8 countries in the sample). Here, $X_{ij}$ represents all the conditional information in the model (i.e. exposures, adjustments, and random effects) and $\phi\geq0$ is a dispersion parameter such that the smaller its value, the smaller the overdispersion is (the limiting case $\phi\to+\infty$ corresponds to the Poisson, i.e. equidisperse, case). Since a logarithm link was assumed, the log of the conditional mean $\mu_{it}$ satisfies

$$\log\left( \mu_{ij} \right)=o_{ij}+ \alpha_{0}+\alpha_{j}+\sum_{k=1}^{3} \beta_{k}{SE}_{ij}^{k}+\sum_{l=1}^{3} \gamma_{l}{BE}_{ij}^{l}+\theta P_{ij},$$

where $o_{ij}=log({LB}_{ij}\cdot U_{ij}/{10}^{5})$is the model offset (${LB}_{ij}$ is the number of live births in the $i$th city and $j$th country, and $U_{ij}$ is the corresponding undercounting correction factor), $\alpha_{0}$ is the model intercept, $\alpha_{j}$ is a country-level random intercept, the $SE_{ij}^{k}$’s are social environment exposures (living conditions, services provision and educational attainment scores), the $BE_{ij}^{l}$’s are the built environment exposures (isolation, population density and mass transit availability), $P_{ij}$ is the total city population and $\beta_{1},\beta_{2},\beta_{3},\gamma_{1},\gamma_{2},\gamma_{3},\theta$ are regression coefficients.

The model setting stated above allows for appropriately modelling the outcome as maternal mortality ratios. Let ${MMR}_{iJ}={10}^{5}\times{(Y}_{ij}/{(LB}_{ij}\cdot U_{ij}))$ the maternal mortality ratio (MMR) at the $i$th city and $j$th country. Then, using basic properties of the logarithmic function, we then have

$$\log\left( {MMR}_{ij} \right)=\log\left( {10}^{5}\times\frac{Y_{ij}}{{LB}_{ij}\cdot U_{ij}} \right)$$

$$=\log\left( Y_{ij} \right)+\log\left( \frac{{10}^{5}}{{LB}_{ij}\cdot U_{ij}} \right)$$

$$=\log\left( Y_{ij} \right)-\log\left( \frac{{LB}_{ij}\cdot U_{ij}}{{10}^{5}} \right)$$

$$=\log\left( Y_{ij} \right)-o_{ij}$$

$$\Longleftrightarrow\log\left( Y_{ij} \right)=\log\left( MMR_{ij} \right)+o_{ij}.$$

Finally, the parameterization we adopt here for the Negative Binomial is

$$P\left( Y_{ij}=n \right)=\frac{\Gamma\left( n+\phi\right)}{\Gamma\left( n+1 \right)\Gamma\left( \phi\right)}\left( \frac{\phi}{\phi+\mu_{ij}} \right)^{\phi}\left( \frac{\mu_{ij}}{\phi+\mu_{ij}} \right)^{n} , n=0, 1, \ldots$$

where $\Gamma\left( x \right):=\int_{0}^{+\infty} s^{x-1}e^{-s}ds$ denotes the Gamma function. This parameterization ensures that the conditional expectation of $Y_{ij}$ given $X_{ij}$ is $E(Y_{ij}|X_{ij}) =\mu_{ij}$ and that the corresponding conditional variance is $V(Y_{ij}|X_{ij}) =\mu_{ij}+\mu_{ij}^{2}/\phi$, so that the overdispersion (relative to the Poisson distribution) is equal to $\mu_{ij}^{2}/\phi$.

**Log-rates clustering analyses model specification**

Our model for the logarithm of mortality ratios is

$$\log\left( {MMR}_{ij} \right)= \alpha_{0}+\alpha_{j}+\varepsilon_{ij}, \varepsilon_{ij}\sim iid N\left( 0, \sigma_{city}^{2} \right),$$

where ${MMR}_{ij}={10}^{5}\times{(Y}_{ij}/{(LB}_{ij}\cdot U_{ij}))$ is the maternal mortality ratio (MMR) at the $i$th city and $j$th country, and $Y_{ij}$, $LB_{ij}$ and $U_{ij}$ are, respectively, the corresponding maternal death counts, number of live births and undercounting correction factor. Here we assume that $\alpha_{j}\sim N\left( 0, \sigma_{country}^{2} \right)$ and that $\alpha_{j}$ and $\varepsilon_{ij}$ are independent of each other for all $i$ and $j$.

In this setting, the total model variance is given by

$$\sigma_{total}^{2}:= \sigma_{city}^{2}+ \sigma_{country}^{2},$$

and therefore the percentage of the variance attributed to each level is, respectively,

$$\sigma_{city}^{2}\left( \% \right):=\frac{\sigma_{city}^{2}}{\sigma_{total}^{2}}\times100\%, \sigma_{country}^{2}\left( \% \right):=\frac{\sigma_{country}^{2}}{\sigma_{total}^{2}}\times100\%.$$

**List of Tables and Figures**

Table S1 provides the definitions and data sources for all variables used in the study. Supplementary Table S2 corresponds to the original Table 1 from the main manuscript and presents the descriptive characteristics of the variables across the 340 cities, stratified by country, was uploaded as a separate supplementary file due to its width (exceeding the 9-column limit for tables in the main article). Supplementary Table S3 contains all the Negative Binomial model results but replied using the CRVS correction factor of WHO (2025) instead of the SALURBAL one used in the main analyses.

Figure S1 shows the correlation matrix between all the exposures used in the paper, Figure S2 contains the scatterplot between log-MMRs and population density, Figure S3 contains a map showing log-MMRs across cities in the sample, and Figure S4 shows the Variance Inflation Factors (VIF) for all the exposures in Model D.

Table S1. Overall structure of the data used in the paper. Each variable was collected and harmonized by SALURBAL’s team based on data from the corresponding source.

| **Variable** | **Type** | **Data Source** |
| --- | --- | --- |
| Maternal mortality counts | Outcome | Vital registration systems |
| % of households with piped water access inside the dwelling | SE exposure (component of living score) | National censuses |
| % of households with more than 3 people per room | SE exposure (component of living score) | National censuses |
| % of population aged 15 to 17 attending school | SE exposure (component of living score) | National censuses |
| % of households with water from a public network | SE exposure (component of services score) | National censuses |
| % of households connected to a public sewage network | SE exposure (component of services score) | National censuses |
| % of population aged > 25 w/ at least secondary education | SE exposure (component of education score) | National censuses |
| % of population aged > 25 w/ at least universitary education | SE exposure (component of education score) | National censuses |
| Area-weighted mean nearest-neighbor distance | BE exposure (isolation) | Global Urban Footprint Project |
| Population density | BE exposure | Worldpop Project |
| Presence of Bus Rapid Transport (BRT) | BE exposure (component of transit) | BRTData and OpenStreetMap |
| Presence of subway system transport | BE exposure (component of transit) | OpenStreetMap |
| Total population | BE exposure (proxy for city size) | Intercensal population projections |
| Live births | Covariate | Vital registration systems |
| SALURBAL undercounting correction factor | Covariate | Bilal et al. (2021) |
| CRVS correction factor | Covariate | WHO (2019) |

| **Table S2:**  Means and standard deviations (in parentheses) of the outcome and exposures by country. 2012-2016 | | | | | | | | | | |
| --- | --- | --- | --- | --- | --- | --- | --- | --- | --- | --- |
| **VARIABLES** | **OVERALL** | **Argentina** | **Brazil** | **Chile** | **Colombia** | **Costa Rica** | **Guatemala** | **Mexico** | **Panama** | **p-value**** |
| Number of cities | 340 | 33 | 152 | 21 | 35 | 1 | 3 | 92 | 3 | – |
| **Outcome** (per 100,000 live births) | | | | | | | | | | |
| Maternal mortality ratio (without correction) | 47.31 | 38.38 | 53.79 | 22.38 | 66.40 | 30.14 | 58.33 | 38.44 | 35.24 | <0.001 |
| [Mean (SD)] | (25.31) | (19.96) | (25.80) | (13.07) | (35.51) | (0) | (10.67) | (12.21) | (16.76) |  |
|  | 51.96 | 38.55 | 55.62 | 22.38 | 102.13 | 38.46 | 58.56 | 38.69 | 40.35 |  |
| CRSV correction***  [Mean (SD)] | (33.11) | (20.06) | 26.71 | 13.07 | 54.67 | 0 | 10.71 | 12.27 | 19.03 |  |
|  | 50.52 | 38.52 | 55.14 | 23.56 | 80.62 | 32.97 | 58.33 | 42.31 | 35.66 |  |
| SALURBAL correction [Mean (SD)] | 31.81 | 20.01 | 27.33 | 14.90 | 62.80 | 0 | 10.67 | 13.21 | 16.49 |  |
| **Social environment features** | | | | | | | | | | |
| Living conditions  [Mean (SD)] | 0.24 | 1.45 | 1.94 | 1.67 | -1.12 | 2.33 | -6.40 | -2.65 | 0.99 | <0.001 |
|  | (2.54) | (0.90) | (1.03) | (0.61) | (1.90) | (0) | (1.78) | (1.98) | (0.37) |  |
| Services provision  [Mean (SD)] | 0.15 | 0.35 | -0.47 | 1.56 | 0.36 | -0.48 | -0.73 | 0.76 | -0.94 | <0.001 |
|  | (1.76) | (0.97) | (2.00) | (0.72) | (1.83) | (0) | (0.50) | (1.26) | (1.17) |  |
| Population educational attainment  [Mean (SD)] | -0.50 | -0.87 | -0.19 | -1.50 | -0.46 | 2.36 | -2.72 | -0.68 | 2.00 | <0.001 |
|  | (1.30) | (0.71) | (1.17) | (0.60) | (1.04) | (0) | (1.41) | (1.53) | (0.95) |  |
| **Built environment features and total population** | | | | | | | | | | |
| Isolation (patches/100ha)  [Mean (SD)] | 97.18 | 99.33 | 89.74 | 95.65 | 107.96 | 65.37 | 85.14 | 106.47 | 73.02 | 0.007 |
|  | (41.62) | (38.41) | (33.04) | (36.32) | (59.61) | (0) | (32.75) | (47.12) | (6.00) |  |
| Population density (hab./km^2^)  [Mean (SD)] | 7,228 | 5,229 | 6,356 | 7,093 | 15,681 | 6,891 | 9,620 | 6,145 | 6,651 | <0.001 |
|  | (3,915) | (1,191) | (2,436) | (1,760) | (4,964) | (0) | (3,017) | (2,248) | (1,235) |  |
| Mass transit availability (%)  [Mean (SD)] | 14.41 | 9.09 | 17.11 | 14.29 | 20.00 | 0.00 | 33.33 | 8.70 | 33.33 | 0.924 |
|  | (35.17) | (29.19) | (37.78) | (35.86) | (40.58) | (0) | (57.74) | (28.33) | (57.74) |  |
| Total population (mi. hab.)  [Mean (SD)] | 0.80 | 0.89 | 0.76 | 0.60 | 0.80 | 2.48 | 1.21 | 0.86 | 0.78 | 0.061 |
|  | (2.08) | (2.65) | (2.05) | (1.35) | (1.55) | (0) | (1.72) | (2.27) | (0.97) |  |

*The standard deviations of (0) here are due to Costa Rica only having one city (San José) in the sample.

**p-value refers to the Kruskal-Wallis test for that variable across countries.

*** The CRVS model: For countries that have a CRVS system, we use a Bayesian CRVS adjustment model to account for errors in reporting of maternal death in the CRVS to obtain the CRVS adjustment factors.

Table S3. Rate ratios of city maternal ratios (computed using the CRVS rather than the SALURBAL undercounting correction factor) associated with a 1SD higher value of social and built environment features.

| **VARIABLES** | **Model A** | | **Model B** | | **Model C** | | **Model D** | |
| --- | --- | --- | --- | --- | --- | --- | --- | --- |
|  | **RR (95% CI)** | **p-value** | **RR (95% CI)** | **p-value** | **RR (95% CI)** | **p-value** | **RR (95% CI)** | **p-value** |
| **Social environment features** | | | | | | | | |
| Living conditions | 0.80 (0.75 – 0.86) | <0.001 | 0.87 (0.80 – 0.96) | 0.003 |  |  | 0.89 (0.82 – 0.97) | 0.009 |
| Services provision | 0.88 (0.84 – 0.91) | <0.001 | 0.93 (0.88 – 0.98) | 0.007 |  |  | 0.93 (0.89 – 0.99) | 0.011 |
| Pop. educ. attainment | 0.92 (0.88 – 0.96) | <0.001 | 0.98 (0.93 – 1.02) | 0.306 |  |  | 0.98 (0.93 – 1.04) | 0.528 |
| **Built environment features** | | | | | | | | |
| Isolation | 1.09 (1.04 – 1.14) | <0.001 |  |  | 1.09 (1.04 – 1.14) | <0.001 | 1.05 (1.01 – 1.10) | 0.020 |
| Population Density | 1.11 (1.04 – 1.19) | <0.001 |  |  | 1.13 (1.06 – 1.21) | <0.001 | 1.11 (1.04 – 1.18) | <0.001 |
| Mass transit availability | 0.96 (0.92 – 0.99) | 0.019 |  |  | 0.94 (0.90 – 0.99) | 0.020 | 0.95 (0.91 – 1.00) | 0.032 |
| Total population | 0.97 (0.94 – 1.01) | 0.184 |  |  | 1.02 (0.97 – 1.08) | 0.430 | 1.04 (0.98 – 1.10) | 0.197 |

RR = rate ratio; 95% CI = 95% confidence interval.

All models are Negative Binomial regressions with maternal mortality ratios (computed using the CRVS rather than the SALURBAL undercounting correction factor) at the outcome, constant dispersion parameter and country- level random intercepts. A logarithmic link was used, and all exposures were standardized to have a mean of 0 and a standard deviation of 1.

Model A: univariate associations.

Model B: multivariate (social environment exposures only) associations.

Model C: multivariate (built environment exposures only) associations.

Model D: full model.
