# Supplementary figures and images for: Association between social and built environment characteristics and maternal mortality in 340 Latin America cities: an ecological study from the SALURBAL study
Source: BMJ Public Health. 2026 Jan 14;4(1):e002437. doi: 10.1136/bmjph-2024-002437 (PMC12815145; doi:10.1136/bmjph-2024-002437)

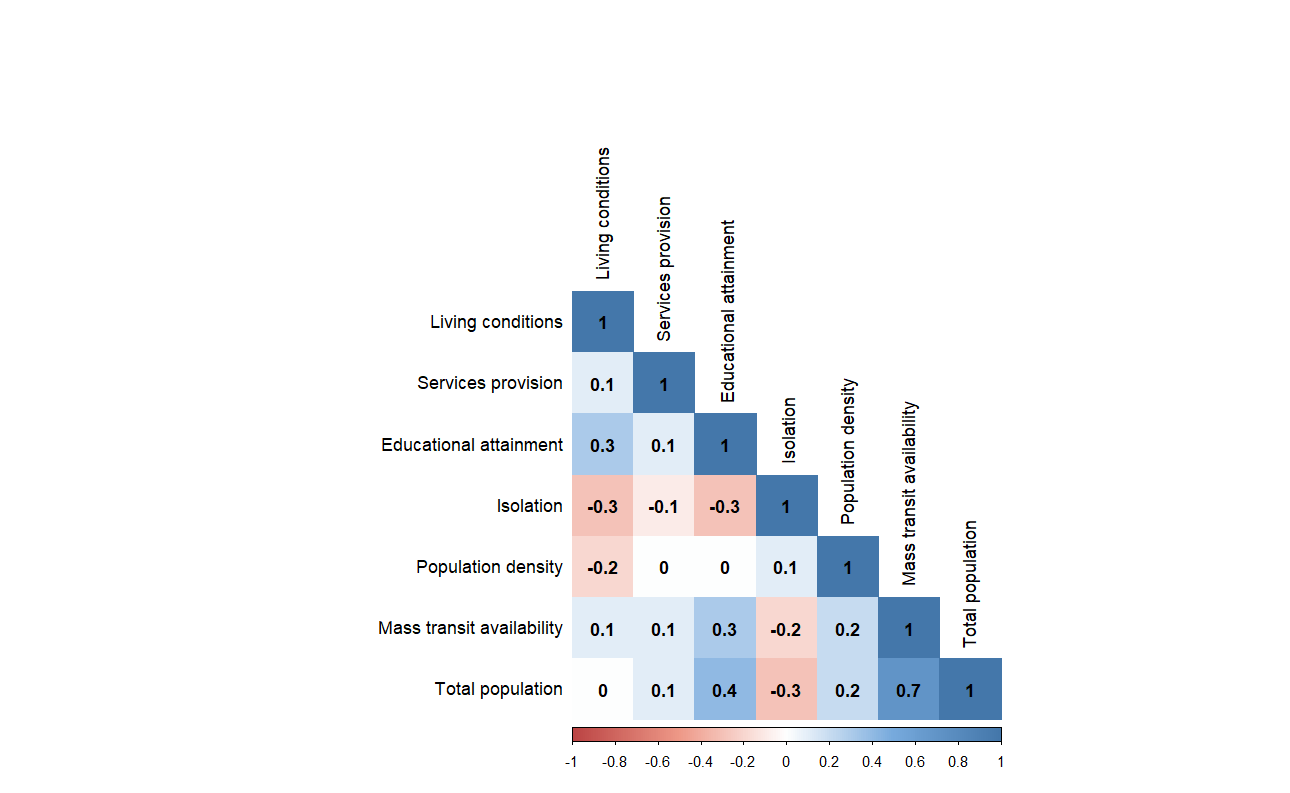


**Figure S1.** Correlation matrix between all of the exposures used in the paper.

Supplement: online supplemental figure 1 [file bmjph-4-1-s002.docx]

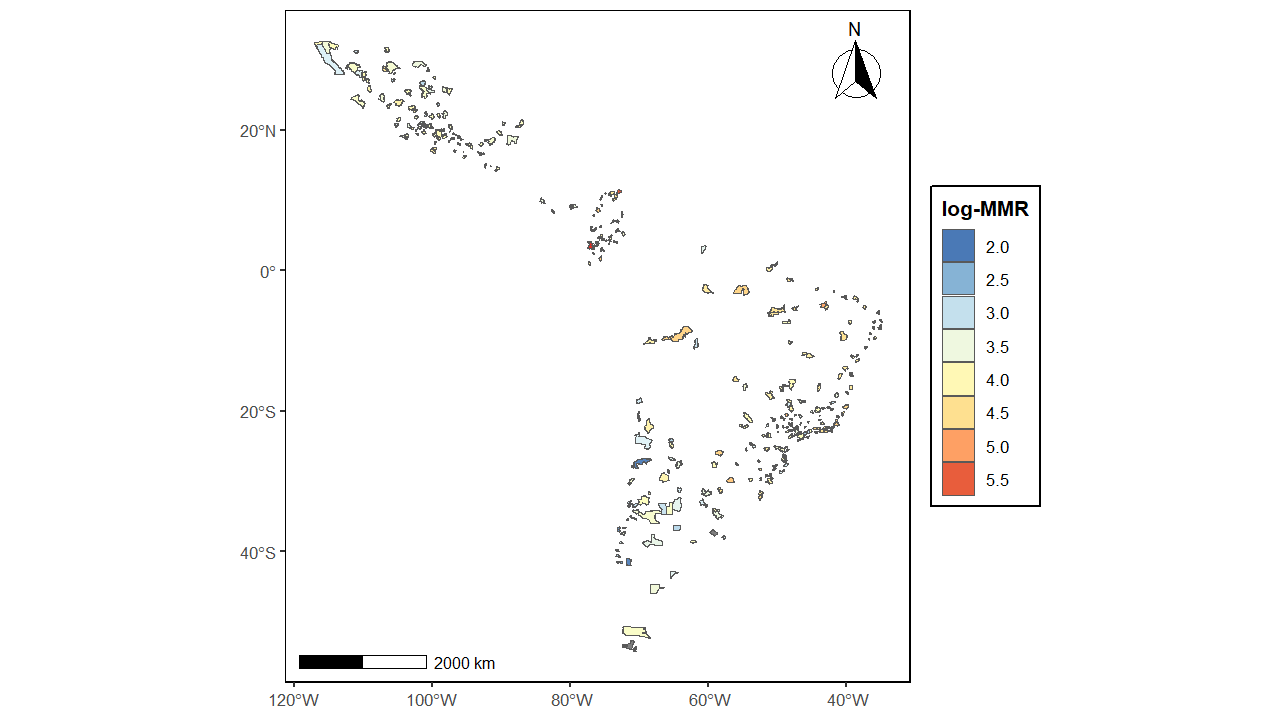


Figure S3. Log-maternal mortality ratios across all cities in the sample.

Supplement: online supplemental figure 3 [file bmjph-4-1-s004.docx]

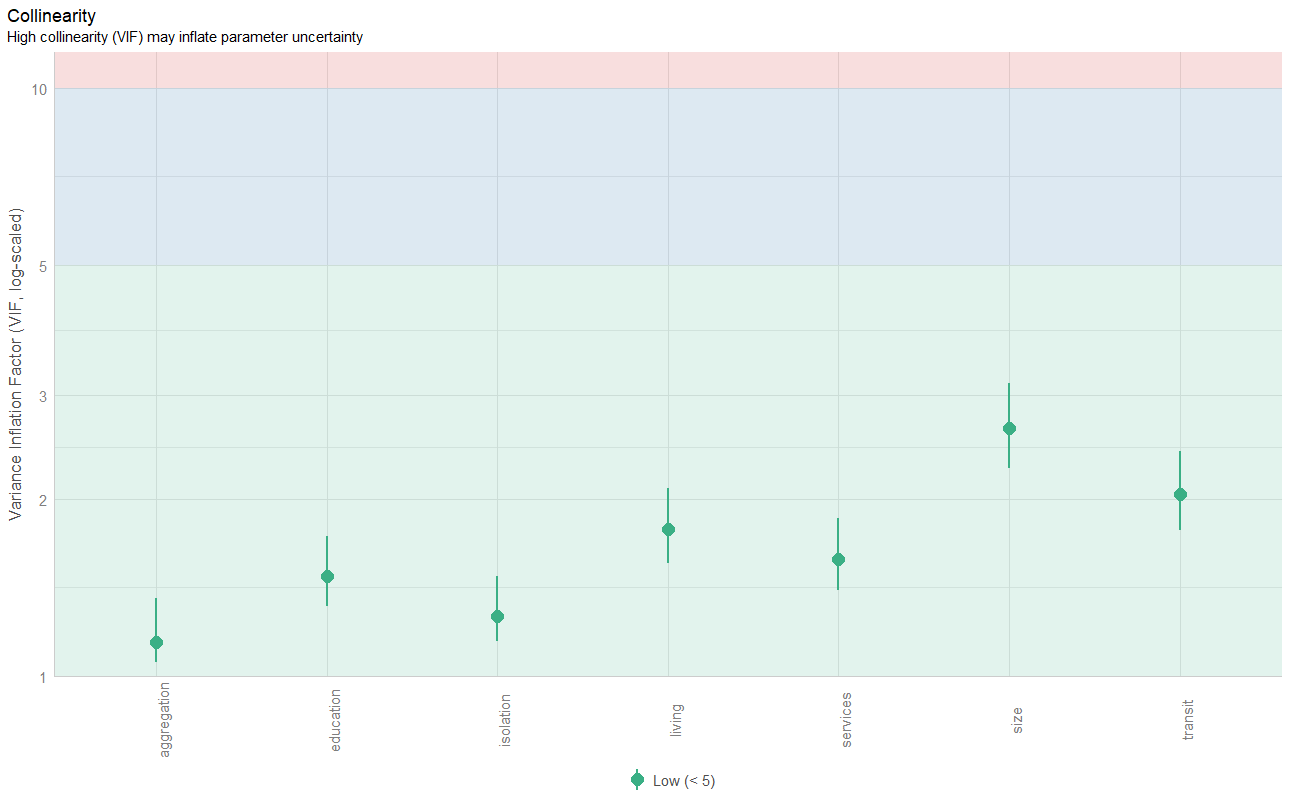
**Figure S4.** Variance Inflation Factors (VIF) for all the exposures in Model D.

Supplement: online supplemental figure 4 [file bmjph-4-1-s005.docx]
