# Supplementary material for: Association between social and built environment characteristics and maternal mortality in 340 Latin America cities: an ecological study from the SALURBAL study
Source: BMJ Public Health. 2026 Jan 14;4(1):e002437. doi: 10.1136/bmjph-2024-002437 (PMC12815145; doi:10.1136/bmjph-2024-002437)
Supplement: online supplemental figure 2 [file bmjph-4-1-s003.docx]

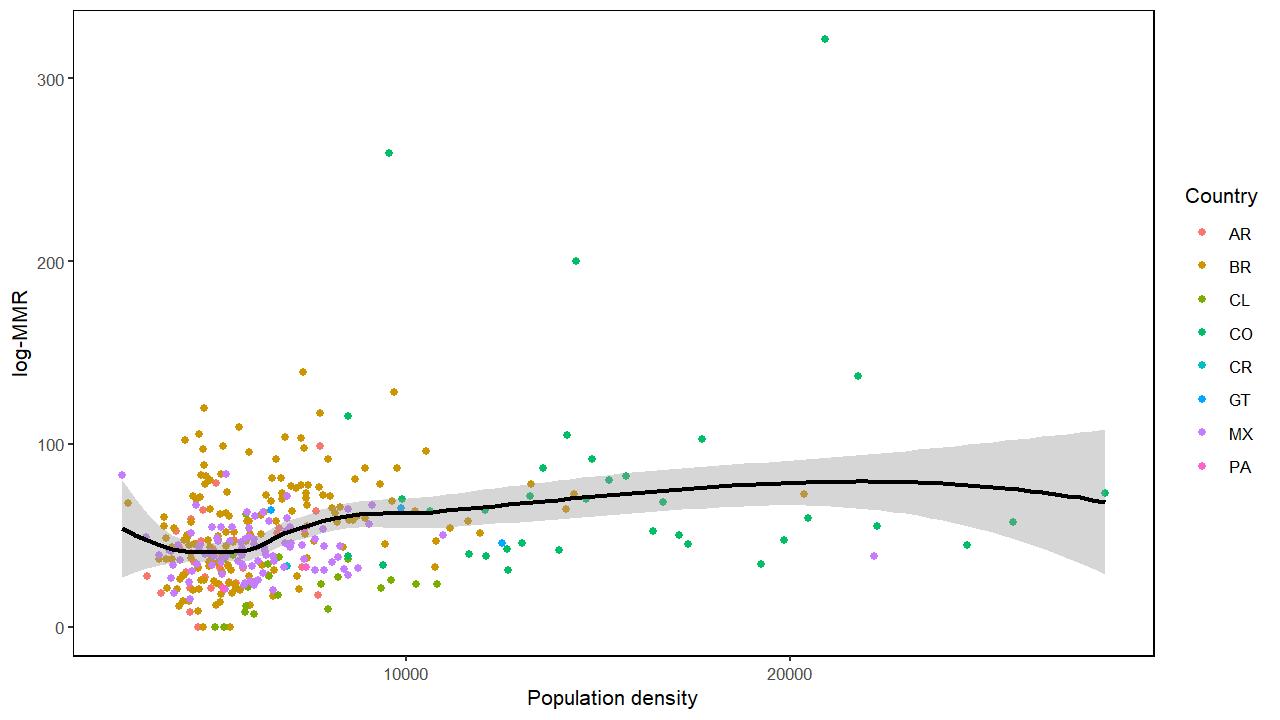


Figure S2. Log-maternal mortality ratios versus population density across the cities in the sample. Black solid line represents the LOESS fit, and the shaded grey area contains the corresponding 95% prediction intervals.
